# Use of 3D printed model as an aid in surgical removal of a rare occurrence of a compound odontome in the anterior mandible associated with impacted teeth

**DOI:** 10.4317/jced.54654

**Published:** 2018-07-01

**Authors:** Neil De Souza, Saurabh Kamat, Paul Chalakkal, Rakshit V. Khandeparker

**Affiliations:** 1 MDS, Lecturer, Department of Pedodontics and Preventive Dentistry, Government Dental College and Hospital, Bambolim, Goa; 2 MDS, Lecturer, Department of Oral surgery, Government dental college, Bambo-lim Goa; 3 MDS, Associate Professor, Department of Pedodontics and Preventive Dentistry, Government Dental College and Hospital, Bambolim, Goa; 4 MDS, Lecturer, Department of Oral surgery, Government dental college, Bambolim Goa

## Abstract

The use of 3D printing in the medical field has been well documented, with significant developments in fabrication of tissues, organs, customized prosthetics, implants, and anatomical models as well as pharmaceutical research. Its use in dentistry, however has been limited mainly to maxillofacial surgery and reconstruction, orthognathic surgery and trauma. Compound odontomes are usually prevalent in the anterior maxilla, however, their occurrence in the anterior mandibular region is rare. This case report highlights the effective usage of 3D printing as an aid in the surgical removal of a compound odontome and impacted incisors in the mandibular anterior region. The surgery was carried out under general anesthesia. A full thickness muco-periosteal flap was reflected and the compound odontome along with the impacted incisors were removed. The defect was restored using a mixture of autogenous scrapes harvested from the chin, xenograft and platelet-rich fibrin. Wound closure was done using 4-0 vicryl. A CBCT scan taken 1 year later confirmed uneventful healing and complete bone regeneration of the surgical defect.

** Key words:**3D printing, model, compound odontome, impacted, incisors.

## Introduction

Three-dimensional (3D) printing is a method of manufacturing 3D objects in layers, either by fusing or by depositing materials such as plastic, metal, ceramics, powders, liquids or living cells (1). In the medical field, 3D printing has been used for the fabrication of tissues and organs, customized prostheses, surgical implants and anatomical models for surgical diagnosis and planning. In the pharmaceutical field, 3D printing has been used for customizing the dosage and delivery of drugs (2,3).

Odontomes may be defined as “tumours formed by the overgrowth of transitory or complete dental tissues” (4). About 22% of all odontogenic tumours are odontomes, which occur as one or many, and may even be associated with missing or impacted teeth (5). This case report highlights the effective usage of 3D printed model as a surgical aid in the removal of a compound odon-tome and impacted incisors from the mandibular anterior region.

## Case Report

**Figure 1:**
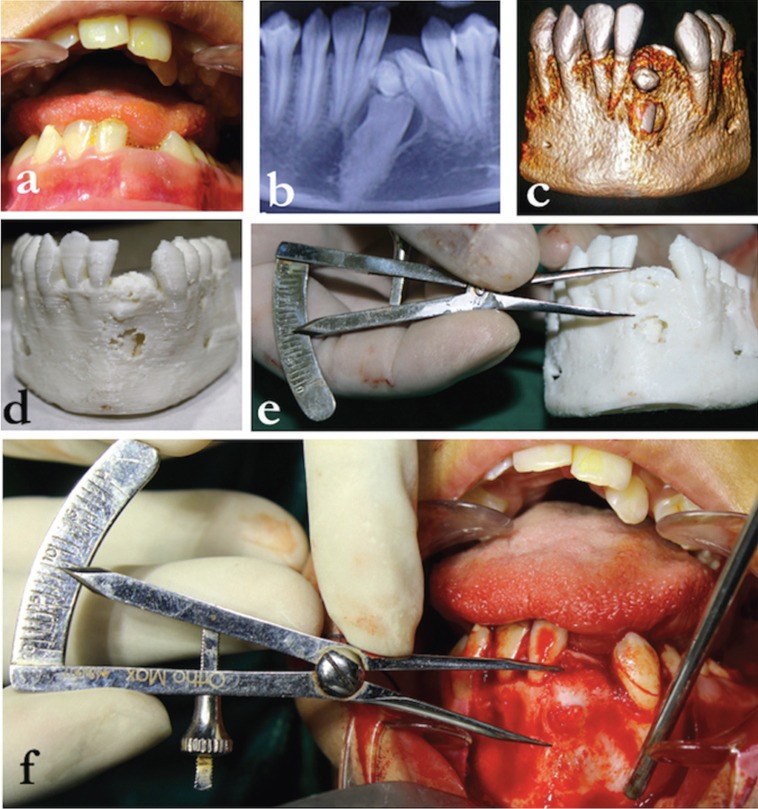
a) Anterior view showing absence of 31 and 32. b) CBCT scan showing cystic involvement with relation to impacted 31, 32 and an odontome. c) CBCT scan showing the presence of impacted 31, 32 and an odontome. d) 3D printed model of the mandible. e) Pre-surgical measurements being made on the 3D printed model. f) Transfer of pre surgical measurements on to the surgical area.

A 12 year old female patient visited the Pediatric dental clinic with the complaint of missing teeth in the anterior region of the jaw (Fig. 1a). Intraoral Examination revealed clinically missing 31, 32 with patient giving no history of previously extracted teeth. A CBCT (Cone beam computerized tomography) scan revealed the presence of impacted 31 and 32, along with the presence of an odontome (Fig. 1b,c). The CBCT image also seemed to suggest a cystic lesion present with relation to the impacted teeth. Hence a joint team of pedodontists, oral surgeons, orthodontics and prosthodontists was consulted to formulate a treatment plan. The treatment plan was divided into two phases: An immediate treatment phase consisting of surgical removal of the impacted teeth with enucleation of the cystic lesion; followed by a long term treatment plan of implant placement and orthodontic correction after the patients growth was complete. The patient and her parents were educated about the surgical procedure, its significant risks and advantages and an informed consent was obtained from the parents for carrying out the procedure. In order to assist in surgical planning, it was decided to obtain a 3D model of the patient’s mandible. A 3D printed model (Fig. 1d) was obtained from the DICOM data of the CBCT scan. The DICOM data was converted into STL file using KISSlicer software and printed using a 3D printer (Medibot Jr ™ by Acton Engineering). A virtual bony window was prepared to expose the cystic lesion associated with the impacted teeth using Osirix software (Fig. 1d). A castroviejo caliper (Ortho Max, India) was used to accurately measure the dis-tance of the impacted teeth from the alveolar crest on the 3d model (Fig. 1e). After all the measurements were recorded, the surgical procedure was undertaken under general anesthesia.

**Figure 2:**
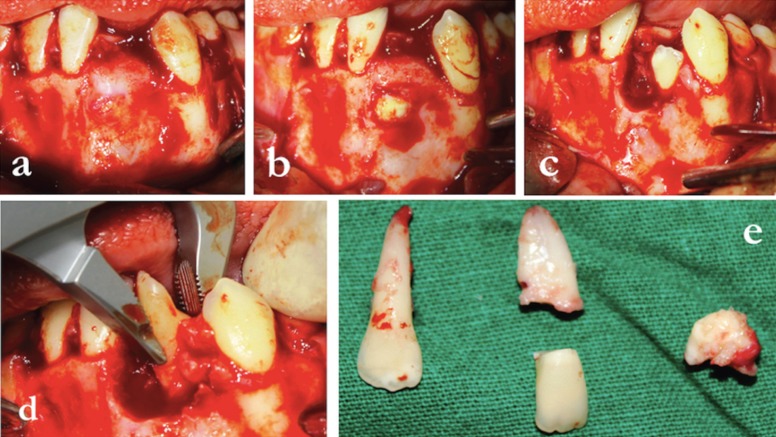
a) Full thickness mucoperiosteal flap raised. b) Surgical exposure of the impacted teeth. c)Extraction site of 31. d) Extraction of 32. e) Extracted 32, 31 (crown and root) and odontome.

**Figure 3:**
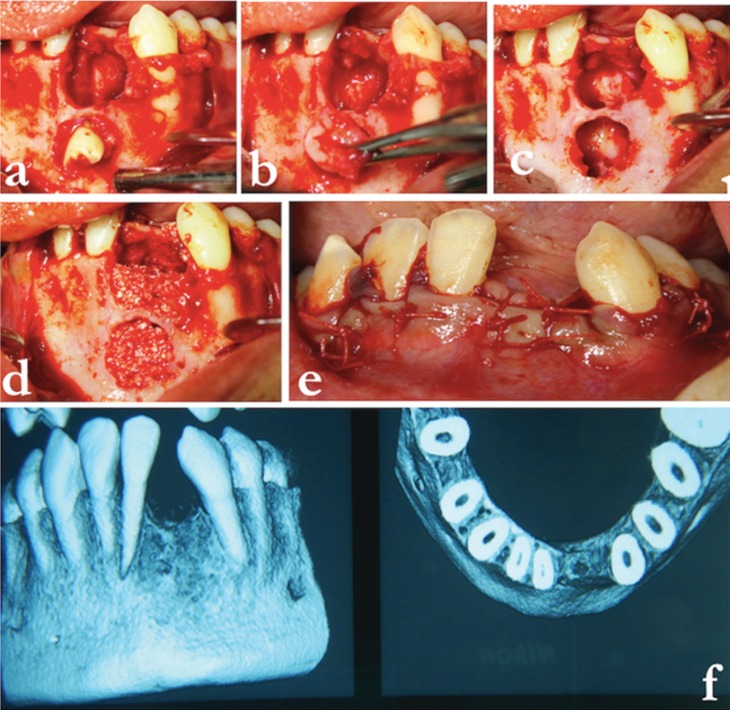
a) Socket containing odontome. b)Enucleation of cystic lesion. c) Extraction sockets after the re-moval of 31,32 and odontome. d) Extraction sites filled with autogenous scrapes, xenograft and PRF. e) Post-surgical sutures. f) CBCT scan taken 1 year post-operatively.

The patient was subjected to oro-tracheal intubation. Following this, 5 ml of lignocaine with 1:100000 adrenalin was infiltrated into the labial vestibule in relation to teeth 33,32,31,41,42 and 43. A crevicular incision was made from the distal aspect of 33 till the distal aspect of 43. On both sides, vertical releasing incisions were given, and a full thickness mucoperiosteal flap was raised (Fig. 2a). As per the measurements made on the 3D model (Fig. 1e), a mini-mally invasive bony window of 5 mm radius was made 10 mm below the alveolar crest (Fig. 1f). The impacted teeth (31 and 32) including the odontome were exposed and surgically re-moved (Figs. 2a-e,3a). Enucleation of the cystic lesion was carried out in toto, followed by thorough curettage of the surgical defect (Fig. 3b,c). Following this, 10 ml of blood was derived from the medial cubital vein of the patient’s left arm. It was then transferred into a test tube (without anti-coagulant) and centrifuged (REMI Model R-8c, India) at 3000rpm for 10 minutes to obtain platelet rich fibrin (PRF). After centrifugation, the test tube showed acellular platelet poor plasma in the top portion, PRF clot in the intermediate portion and red blood cells at the bottom portion. PRF was removed from the test tube with the help of a sterile tweezer and placed in a dappen dish. The post-surgical defect was filled with a combination of autogenous scrapes harvested from the chin, xenograft (Geistlich Bio-Oss®) and PRF (Fig. 3d). Wound closure was done using 4-0 vicryl (Fig. 3e), following which the patient was extubated une-ventfully. Regular follow ups done at 1 week, 1 month and 3 months showed uneventful wound healing. A CBCT scan taken 1 year later confirmed sufficient regeneration of bone at the site of the defect (Fig. 3f). The second long term phase of treatment of implant place-ments followed by orthodontic space closure has been planned for after the growth of the pa-tient is complete.

## Discussion

In dentistry, 3D printing has been used for maxillofacial surgery and reconstruction,6 orthog-nathic surgery (7) and trauma (8). They can be obtained from 2 dimensional images such as x-rays, magnetic resonance imaging and computerized tomography scans (9,10). The various methods for 3D printing include selective laser sintering, thermal inkjet printing, stereolithography, direct metal laser sintering, laminated object manufacturing, electron beam melting and fused deposi-tion modeling (FDM) (10-12). In an FDM printer, beads of heated plastic (acrylonitrile butadiene styrene) are released from the printhead as it moves, eventually building up the model in thin layers, equal in size with the real object (11,13). Since the material is liquefied as it extruded, it fus-es and bonds to the layer beneath (13). Eventually each layer of plastic cools and hardens to form a solid object (11).

Trauma, infection and loss in genetic control have been regarded as possible etiologic factors in the occurrence of odontomes (4,5). They have also been associated with syndromes like gardner’s syndrome, hermann’s syndrome and basal cell nevoid syndrome (14). However, the patient had not reported of any previous trauma to the mandible, neither did she have a syndrome or any other medical condition. A compound odontome is composed of enamel, dentin and cementum, but may present a lobulated appearance. Compound odontomes are generally found in the inci-sor-cuspid region of the maxilla (61%), however, complex odontomas are commonly found in the premolar-molar region of the mandible (34%) (12). Based on the above features, we confirmed it to be a case of compound odontoma associated with impacted incisors (31 and 32), with a rare occurrence in the mandibular anterior region.

In this case, 3D printing technology assisted us in the following ways: 1. The mandibular mod-el functioned as a pre surgical tool from which we could obtain the precise location of the odon-tome and the impacted incisors with the help of measurements. 2. The 3d printed model helped in patient and parent eduction and awareness of the condition. 3. Pre-surgical measurements and planning helped reduce the the surgical time and aided in the ease of performing the sur-gery.
